# The fingerprint method for analysis of thermal desorption spectra

**DOI:** 10.1038/s41529-025-00718-z

**Published:** 2025-12-10

**Authors:** Philipp Hammer, Oleg E. Peil, Ahmad Azizpour, Liese Vandewalle, Kim Verbeken, Tom Depover, Vsevolod I. Razumovskiy

**Affiliations:** 1 Christian Doppler Laboratory for digital material design guidelines for mitigation of alloy embrittlement, Materials Center Leoben Forschung GmbH, Vordernberger Straße 12, 8700 Leoben, Austria; 2 Materials Center Leoben Forschung GmbH, Vordernberger Straße 12, 8700 Leoben, Austria; 3 Department of Materials, Textiles and Chemical Engineering, Ghent University, Technologiepark-Zwijnaarde 46, 9052 Zwijnaarde, Belgium

**Keywords:** Materials science, Physics

## Abstract

The analysis of thermal desorption spectra (TDS) and the calculation of hydrogen detrapping activation energies rely on Gaussian peak deconvolution and Choo-Lee plot regression since 1982. However, this method imposes important assumptions about the number and shape of the TDS peaks used for fitting. In this study, we propose the *fingerprint* method, an alternative approach that eliminates these long-standing constraints. By applying the *fingerprint* analysis to eight TDS spectra from three different Fe-C model alloys, we demonstrate its exceptional sensitivity and ability to resolve activation energy distributions – the material *fingerprint* – unattainable with traditional methods. We further showcase by manual and automated analysis how the such obtained *fingerprints* can be used to uniquely distinguish the TDS spectra of each alloy independent of the heating rate. Thus the *fingerprint* method also increases experimental efficiency by reducing the amount of necessary heating rates for TDS down to one.

## Introduction

The advancement of materials for the emerging hydrogen energy sector relies on obtaining accurate data regarding hydrogen concentration, distribution, and detrapping activation energies. This information is crucial for optimizing hydrogen storage capacity and enhancing resistance to hydrogen embrittlement. Thermal desorption spectroscopy (TDS) stands out as a pivotal experimental technique for the precise analysis of these hydrogen-related properties in metallic materials. In TDS, a hydrogen pre-charged specimen is heated at a constant rate, and the desorbing hydrogen is measured via a mass spectrometer, resulting in a characteristic spectrum with one or multiple peaks. Traditionally, Gaussian peak deconvolution and Choo-Lee plot regression^1^ have been the most common approaches for interpreting TDS spectra since 1982. However, these methods suffer from significant assumptions, particularly regarding the number and shape of the TDS peaks.

Choo and Lee^1^ adopted Kissinger’s theory to determine the detrapping activation energy of hydrogen from thermal desorption spectra. In this approach, the $ln\left(\phi /{T}_{c}^{2}\right)$ is plotted vs. $\left(1/{T}_{c}\right)$ for each peak with position *T*_*c*_ and multiple heating rates *ϕ* to obtain the detrapping activation energy *E*_*a*_ from the slope of the resulting Choo-Lee plot, which equals − *E*_*a*_/*R*, where *R* is the ideal gas constant. While this method works well for spectra with single or clearly separated peaks, the challenge of deconvoluting spectra into multiple subpeaks leads to significant difficulties^2^. Traditionally, spectra are fitted with multiple Gaussian peaks, which are then analyzed using the Choo-Lee method to determine their individual detrapping activation energies. These energies are then associated with specific crystal lattice defects (such as dislocations or vacancies) or microstructural features (like grain boundaries or precipitates). However, Kissinger^3^ pointed out that reaction-rate peaks are asymmetrical, and Drexler et al.^2^ have shown that even strong correlation coefficients in the Choo-Lee plot do not guarantee correct deconvolution, resulting in a potentially ambiguous interpretation of the obtained detrapping activation energies.

The interpretation of thermal desorption spectra has been refined ever since by comparing the determined detrapping activation energies with ab-initio density functional theory (DFT) calculations or fitting the spectra with physical diffusion models based on the works of Oriani^4^ and McNabb and Foster^5^. While DFT can address various trapping sites within almost any crystal structure, it is usually limited by the size of the considered atomic structures and the number of configurations necessary to calculate a complete set of trapping energy distributions. Therefore, detailed calculations of trapping at defects in chemically complex systems like alloys often become a computationally unfeasible task. On the other hand, fitting thermal desorption spectra with diffusion models may allow retrieving not only the trapping energies but also the associated trap densities^2,6^. However, like the Gaussian deconvolution procedure, this approach has the disadvantage of requiring prior knowledge of the number of traps and the associated trapping energies (e.g., from DFT), i.e., the number and the initial position of peaks used for the spectra deconvolution^2,6–9^.

Representation of a complex material microstructure by a fixed number of “effective” traps, each characterized by specific (average) detrapping activation energies, significantly influences our understanding and interpretation of hydrogen-material interactions in nature. Every material microstructure offers a multitude of hydrogen traps characterized by a distribution of trapping energies rather than by a single “effective” energy, which is difficult (if not impossible) to define for a general case. For example, grain boundaries and dislocations offer multiple sites to trap hydrogen in their vicinity, each slightly varying in their interaction energy with hydrogen^10^.

To address these methodological shortcomings, we propose a new *fingerprint* method for TDS analysis, whereby a TDS spectrum is mapped onto a hydrogen distribution over a continuous spectrum of traps with varying energies, uniquely characterizing the material microstructure (see also method comparison in Fig. 2). This mapping is performed with the help of the distributed activation energies model (DAEM) – originally put forward to describe relaxation processes in vapor-deposited metallic films^11^ and currently used for analysis of temperature programmed desorption of chemical reactions and other diffusion-kinetic processes^12–18^. This implementation of DAEM extends the assumption of first-order reaction kinetics for each peak in the original Gaussian deconvolution procedure to an extensive number of parallel first-order reactions with distinct activation energies and frequency factors^13–17,19,20^. With an increasing number of independent reactions, their activation energies eventually converge to a continuous distribution function – i.e., the spectral *fingerprint* of the material microstructure.

**Fig. 1:**
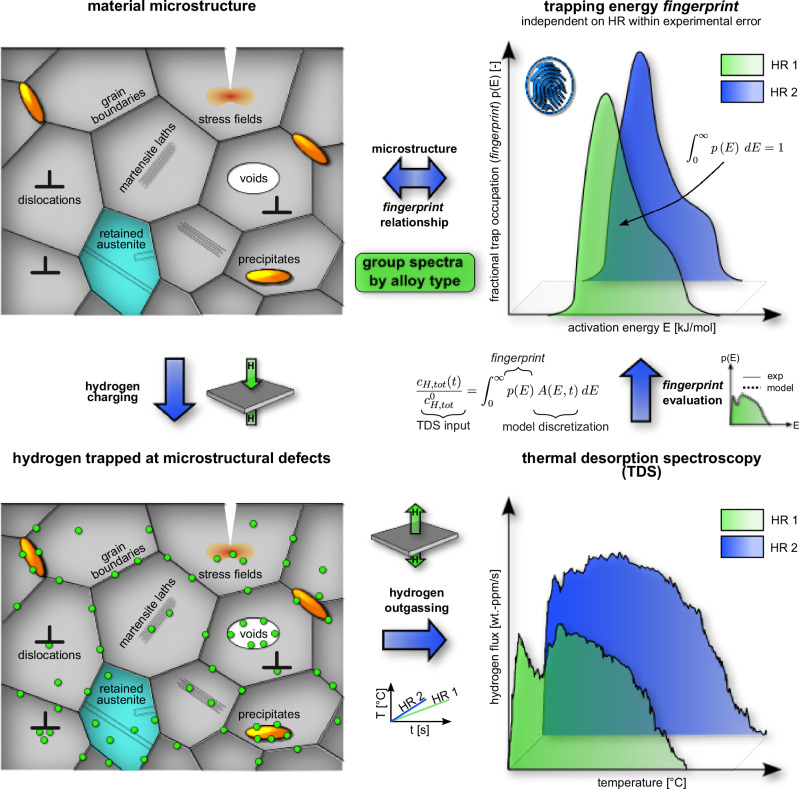
Workflow for the *fingerprint* method. Overview of the *fingerprint* method and how the trap state of hydrogen in the material microstructure can be linked to the *fingerprint* obtained from the TDS spectrum. Note that since only traps populated by hydrogen are captured by the TDS measurement, only those traps are reflected in the *fingerprint* as well. Importantly, TDS measurements obtained from a hydrogen saturated material result in identical *fingerprints* within experimental error, irrespective of the used heating rate.

The basic idea of our approach is sketched in Fig. 1. A material with various hydrogen-trapping microstructure features (top left) is charged until traps are fully saturated (bottom left). The distribution of hydrogen over the traps is probed by TDS measurements, whose resulting spectra (bottom right) strongly depend on the microstructure and experimental conditions (heating rate). By separating these two factors and by converting the thermal desorption spectra into activation-energy distributions, we obtain the *fingerprint* of the original microstructure (top right). In the following, we describe this workflow in more details and present several examples demonstrating its sensitivity in identifying unique material-specific activation-energy distributions on a qualitative level.

**Fig. 2:**
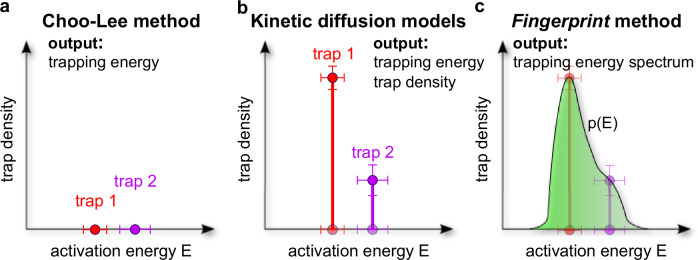
A schematic representation of qualitative differences in terms of trap density and trap activation energy analysis between methods for TDS interpretation. **a** The Choo-Lee method^1^, **b** kinetic diffusion models^4,5^, and **c** the *fingerprint* method.

Before proceeding to the main results, we would like to show a schematic side-by-side comparison of our method (Fig. 2c) with the most commonly employed methods for interpreting TDS data, i.e. the Choo-Lee method^1^ (Fig. 2a) and kinetic diffusion modeling approaches^4,5^ (Fig. 2b). The comparison is framed in terms of trap activation energy and trap density (the coordinates of the trapping energy *fingerprint*) which enables a clear explanation of each method’s limitations and capabilities. Specifically: **a** the Choo-Lee method can determine trapping energies, provided the number of associated traps is known; **b** kinetic diffusion models offer the advantage of yielding both trapping energy and trap density values for a predefined number of traps; and **c** the *fingerprint* method delivers a comprehensive spectrum of trapping energies and densities. Once quantified, the features of the calculated *fingerprints* should be reflective of the average detrapping activation energies obtained by other methods.

Finally, while the fingerprint method is demonstrated here primarily as a tool for sorting unknown TDS spectra, its principal value lies in the additional insights it provides into hydrogen trapping states within the material (as demonstrated in Fig. 2), as well as in improving the efficiency of TDS data interpretation. Together, these advantages are expected to contribute to a deeper understanding of hydrogen embrittlement and to accelerate the development of materials designed to mitigate its effects.

## Results

### From TDS to the *fingerprint*

The *fingerprint* method is based on the assumption that the TDS flux can be considered as if it was generated by a linear composition of parallel first-order reactions, each characterized by its own activation energy, *E*^13–17,19,20^. Introducing a conversion factor, *g*(*t*) = *c*(*t*)/*c*(0), where *c*(*t*) is the total amount of hydrogen in the material at time *t*, we can find it as a weighted average over individual reactions,

1 $$g(t)=\mathop{\int}\nolimits_{0}^{\infty }p(E)A(E,t)\,dE,$$

with *p*(*E*) being the initial distribution of hydrogen over reactions with activation energy *E* and *A*(*E*, *t*) is a material-independent function representing partial conversion factors (see Methods for details)^14,16,17,21^. An important aspect of this formulation is that it provides a separation of the total conversion factor into a material-specific part, *p*(*E*), and a part characterizing the experimental setup, *A*(*E*, *t*).

The above equation can be considered as an integral transform of the initial distribution *p*(*E*) to the total hydrogen content, with the kernel, *A*(*E*, *t*) describing detrapping kinetics of individual reactions. The total hydrogen content is related to the hydrogen flux, *ϕ*(*t*), observed in TDS as $c(t)=c(0)-\mathop{\int}\nolimits_{0}^{t}\phi (t^{\prime} )\,dt^{\prime}$. Another important assumption is that *p*(*E*) is related only to material microstructure, being independent of particular TDS measurement details, *provided that* the measurement is performed on samples fully saturated with hydrogen. *p*(*E*) can thus be considered as a unique *fingerprint* of the material, which can be obtained from a given TDS spectrum by inverting the above integral transform.

**Fig. 3:**
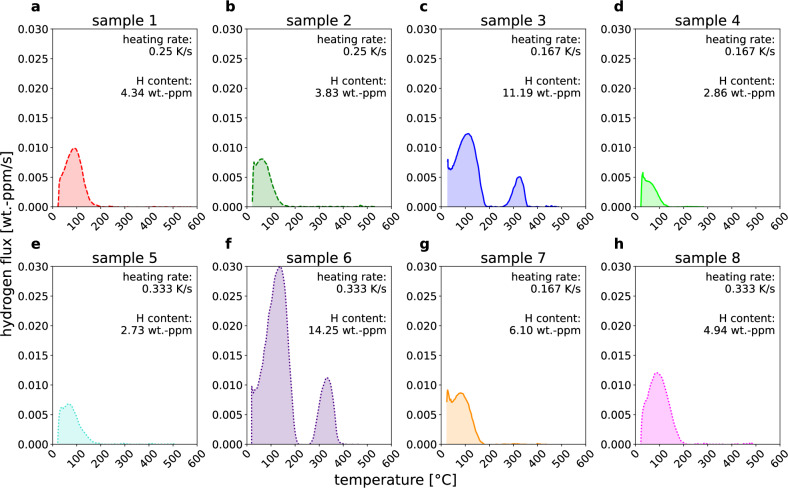
TDS spectra of samples 1 to 8. **a**–**h** show the measured TDS spectra of samples 1 to 8 in order as they enter the data pool for the *fingerprint* analysis.

As a showcase, we have received a total of eight TDS spectra measured by the research group of Ghent University on samples taken from an a priori unknown number of alloys, with the goal to determine the number of alloy types and to group TDS spectra by alloy type. All samples were charged with hydrogen to saturation and subjected to up to three different heating rates to record their thermal desorption spectra. Fig. 3 displays the TDS measurement results of all eight samples. The total hydrogen concentration displayed on each spectrum is calculated as the time integral of the TDS spectra. The results are assigned to sample 1-8 and appear as they enter the data pool for the *fingerprint* method analysis, i.e., without prior knowledge of the alloy behind each measurement. One can immediately spot a difference between sample 3 and 6 with the rest of the spectra, however there is little qualitative difference in the shape of the remaining TDS spectra, making identification of the underlying alloys less trivial, creating a perfect testing ground for our newly proposed method.

**Fig. 4:**
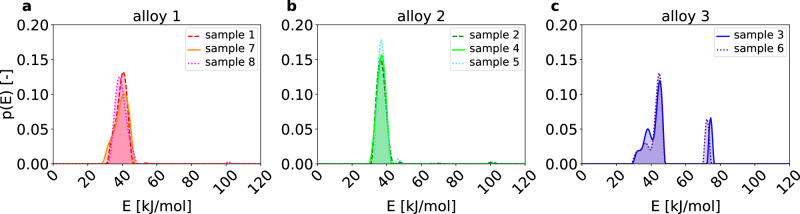
*Fingerprints* grouped by alloys. The hydrogen activation energy *fingerprint* for **a** alloy 1 (sample 1, 7, 8), **b** alloy 2 (sample 2, 4, 5), and **c** alloy 3 (sample 3, 6). Samples 1 to 8 are grouped into three alloys based on the appearance of their respective *fingerprints*.

This method can be directly applied to the analysis of the TDS spectra shown in Fig. 3. The associated *fingerprints*, displayed in Fig. 4, represent the distribution *p*(*E*) of hydrogen atoms populating traps of activation energy *E* at the start of the TDS measurement (*t* = 0). The area under the peaks in Fig. 4 reflects the fraction of hydrogen atoms “trapped” at crystal structure defects within specific energy intervals (characteristic trapping energies). Here, it has to be mentioned that only hydrogen traps that initially contain and later release hydrogen upon heating during the TDS measurement can be included in the analysis, and therefore it is essential that the investigated material is saturated with hydrogen prior to the TDS measurement. On the contrary, the variations in the heating rate have only a minor influence on the *fingerprints*, thus resulting in nearly identical *fingerprints* within experimental accuracy for the same alloy independent of the heating rate used to perform the TDS. This heating rate independence is schematically depicted in Fig. 1 and becomes evident when comparing the grouped *fingerprints* obtained from different heating rates in Fig. 4. Importantly, it also marks a potential efficiency increase of the *fingerprint* method over conventional methods, where experiments on at least three different heating rates have to be conducted in order to calculate hydrogen detrapping energies.

### Visual *fingerprint* analysis

The initial visual analysis, in the spirit of Fig. 2, of the *fingerprint* feature shown in Fig. 4 clearly reveals three distinct groups of spectra (Fig. 4a–c) that are closely related, with their respective *fingerprint* main peak maxima appearing within the uncertainty of ≈ 3 kJ/mol. Thus, it can be reasoned that a total of three different alloys are present in the data pool, which are labeled alloy 1 to 3 in ascending order of their first appearance in the dataset. Consequently, samples 1, 7, and 8 from Fig. 3a are ascribed to alloy 1, while samples 2, 4, and 5 are labeled as alloy 2, and samples 3 and 6 as alloy 3.

A posteriori, it is confirmed by the research group of Ghent University that the TDS spectra indeed belong to three distinct Fe-C alloys of increasing C-content, as previously characterized by Pinson et al.^22,23^. TDS spectra herein ascribed to alloys 1, 2, and 3 belong to Fe-0.4C, Fe-0.2C, and Fe-1.1C, respectively^22^. The *fingerprints* for alloy 1 exhibit a primary peak with the peak maxima ranging from 38.5 to 41.5 kJ/mol and a shoulder appearing on the left side of the main peak. Similarly, alloy 2 displays a main peak within the energy range of 36.5 to 37 kJ/mol for its maxima and a small satellite peak to the right of the main peak. This indicates that two main trap sites are present in both alloys 1 and 2, with their respective trapping energy distributions partly overlapping. Despite significant changes in the hydrogen concentrations for individual samples (see Fig. 3) originating from natural experimental variations in microstructure and hydrogen charging, the *fingerprints* of even closely related alloys (Fe-0.2C and Fe-0.4C) could successfully be assigned to their respective alloys based on their shape and main peak maxima as the sample-to-sample variations are smaller than the variations due to different material microstructure. Consistent with this finding, Pinson et al.^22^ have determined dislocations (24.1–27.7 kJ/mol) and high-angle grain boundaries (37.4–38.2 kJ/mol) as trap types for these alloys. While the main goal of this work is the qualitative introduction of the *fingerprint* method and a rigorous quantification of the calculated hydrogen activation energies is yet to be done, the obtained results are already remarkably close to the classical analysis conducted within ref. ^22^.

Qualitatively, both the TDS spectra of samples 3 and 6 (Fig. 3c, f) as well as their respective *fingerprints* (Fig. 4c) are quite different from those belonging to alloys 1 and 2. While the main *fingerprint* peak appears to consist of three subpeaks, there is also an additional peak appearing in the energy range from 72 to 74.5 kJ/mol. Again, the prediction of the *fingerprint* method is qualitatively consistent with the conventional analysis, as Pinson et al.^22^ found dislocations (18.0 kJ/mol), cementite (26.1 kJ/mol), and high-angle grain boundaries (40.7 kJ/mol) as trap types for the low-temperature peak in the TDS. Furthermore, the high C-content in the Fe-1.1C alloy from refs. ^22,23^ leads to the formation of retained austenite within the martensite matrix after quenching. Consequently, the high-energy peak for alloy 3 in Fig. 4c can be associated to the transformation of retained austenite and the release of hydrogen trapped therein^22,23^.

### Automated *fingerprint* analysis

The simplified visual analysis of the peak maxima obtained by the *fingerprint* method results presented above offers a straightforward approach to analyzing TDS data that can intuitively be compared to the average detrapping activation energies obtained externally (see Fig. 2). However, this form of analysis is not rigorous enough for an unambiguous interpretation of the results. Therefore, in what follows, we would like to present a fully automated approach, based on *fingerprints*, to classification of TDS data according to the alloy type.

The calculation of the *fingerprint p*(*E*) is based on the least squares optimization between the measured and predicted TDS spectra in their integrated form (see section 4 Methods). For a given total hydrogen concentration and heating rate, the activation energy distribution of a specific TDS spectrum (i.e., its *fingerprint*) can be straightforwardly applied to calculate a TDS spectrum for any other hydrogen concentration and heating rate (section 4). Thus, the *fingerprint* obtained from each sample in Fig. 4 can be used to recalculate a TDS spectrum for any other sample at any heating rate.

**Fig. 5:**
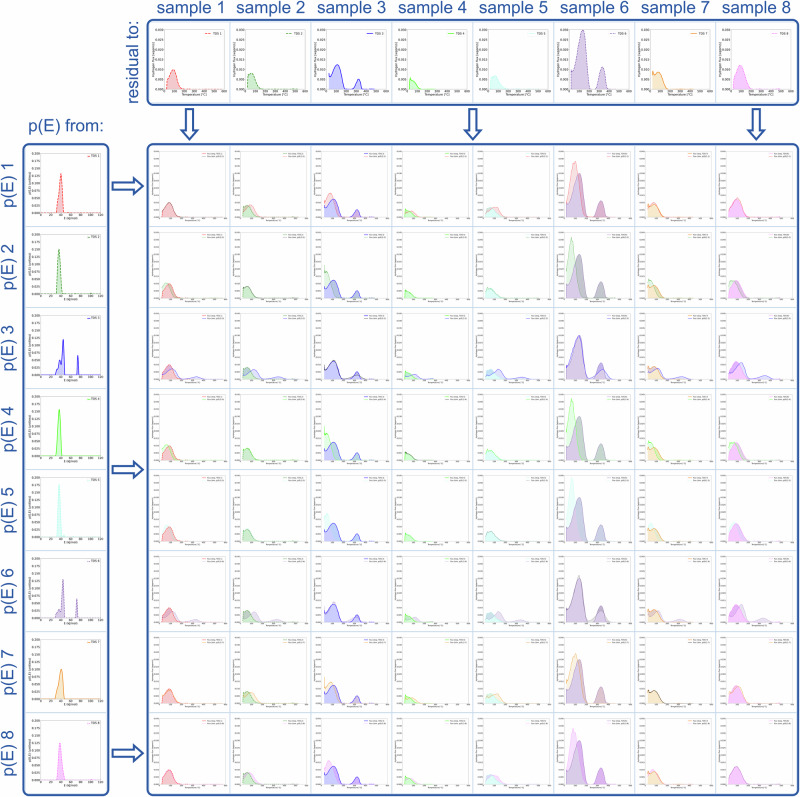
Cross-validation matrix for the automated *fingerprint* analysis. Predicted TDS spectrum (row color and linestyle) by applying the *fingerprint* (i.e. p(E)) of the sample in the row to the heating rate and total hydrogen concentration of the sample in the column, compared to the measured TDS (column color and linestyle).

This process results in the cross-validation matrix displayed in Fig. 5, which compares the predicted hydrogen flux from the applied *fingerprint* (style and color from the *fingerprint* to the left of each row) to the actual measurement (style and color from the TDS spectrum on top of each column). Assuming that the obtained *fingerprint* is unique for each alloy, the predicted hydrogen flux from the *fingerprint* of one sample should satisfactorily reproduce the TDS spectra of the samples belonging to the same alloy, while the fit to other samples should be less accurate. Indeed, it can be observed on the diagonal in Fig. 5 that the *fingerprint* very accurately predicts the hydrogen flux of the TDS spectra from which it is derived.

**Fig. 6:**
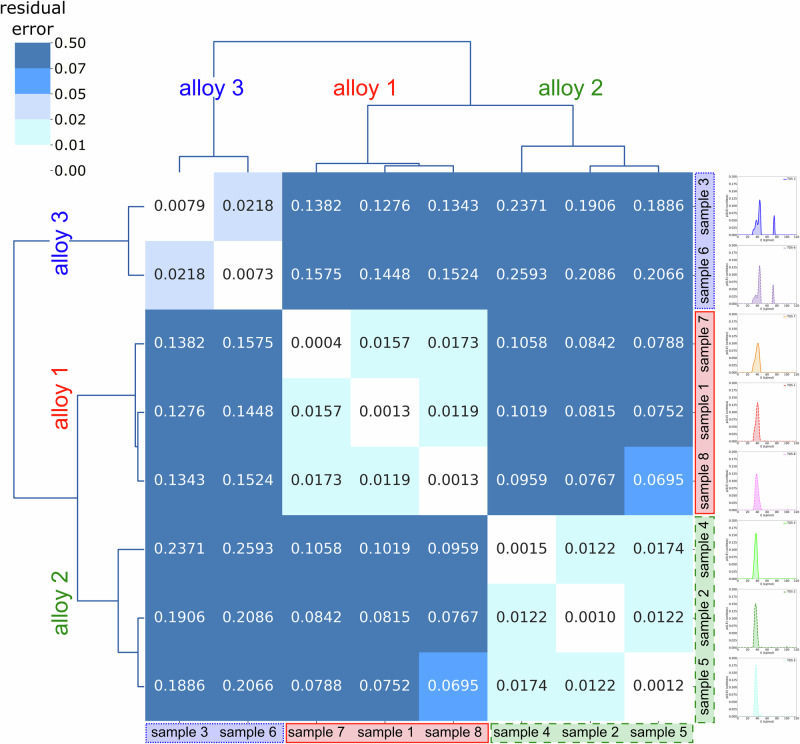
Cluster map with a dendrogram of the residual root-mean-square differences between TDS spectra calculated from the *fingerprints* and the experimentally measured data. For the visual comparison, see Fig. 5. The length of the branches represents the average Euclidean “distance” (i.e. similarity) to each other^24,26,27^. The results of this calculation uniquely assign TDS from sample 1, sample 7, and sample 8 to alloy 1; sample 2, sample 4, and sample 5 to alloy 2; sample 3 and sample 6 to alloy 3.

To quantify the accuracy with which the *fingerprint* of one sample can be applied to others, the residual root-mean-square error of the measured and predicted retained hydrogen fraction is calculated for all combinations shown in Fig. 5 (see Equation (22), Equation (25), and Equation (28) in Methods). The root-mean-square error can be used as a natural, heating-rate invariant, metric for comparing TDS spectra. By applying this metric to our case, we have obtained cross-validation scores to sort out available TDS data according to the alloy they have been measured on, based on the calculated activation energy *fingerprints*. Fig. 6 shows the resulting matrix of residual root-mean-square errors between the predicted and the measured TDS spectra. The results are clustered by color into groups of similar residual errors and connected with the help of a dendrogram. The dendrogram provides a more detailed cluster analysis, as the branch lengths correspond to the average Euclidean distance between the calculated errors^24–27^.

The cluster analysis unambiguously identifies three groups of alloys: samples 1, 7, and 8 belonging to alloy 1; samples 2, 4, and 5 belonging to alloy 2; and samples 3 and 6 belonging to alloy 3. In other words, the *fingerprints* of samples 1, 7, and 8, which belong to alloy 1, can accurately reproduce each other’s TDS spectra. The same is true for the groups of samples 2, 4, and 5 of alloy 2 and samples 3 and 6 of alloy 3, respectively, confirming the assumption of the uniqueness of the *fingerprint* for every considered alloy. This result fully coincides with the visual analysis presented in Fig. 4, with the added advantage of being a fully automated procedure that does not require human supervision. The dendrogram analysis further indicates that alloys 1 and 2 exhibit very similar trapping behavior, whereas alloy 3 is less closely related to the former two. Thus, the cluster analysis integrated with the *fingerprint* method not only accurately groups the measurements by their respective alloys but also identifies that alloy 3, which compared to alloys 1 and 2 contains cementite and retained austenite as additional microstructural elements, can be clearly distinguished from those alloys which have a fully martensitic microstructure^22,23^.

## Discussion

So far, the described model marks a significant improvement to the standard Gaussian deconvolution of TDS spectra as it does neither need presumptions on the number of traps nor on the shape of a fitting function. Instead, it provides the (fractional) hydrogen distribution over a continuous energy range, which arguably much closer reflects the real microstructure in a material. Nevertheless, the present implementation of the *fingerprint* approach relies on certain assumptions that need to be examined to understand possible limitations of interpretation.

First, the underlying model is based on the same first-order reaction kinetics as Choo and Lee^1^, which do not explicitly describe hydrogen diffusion within the sample. Although this may complicate the analysis in cases involving large sample thicknesses or sluggish hydrogen diffusion, this assumption has been verified for the considered cases of TDS measurements done on low carbon steels with the standard sample thickness of 1 mm. Two other important aspects, are: (i) the choice of the frequency factor *ω* in the kinetic kernel *A*(*E*, *t*) (see Equation (19))^13,16,17,20,21,28,29^ and (ii) the model sensitivity to noise in the measurements^12,14–16^.

**Fig. 7:**
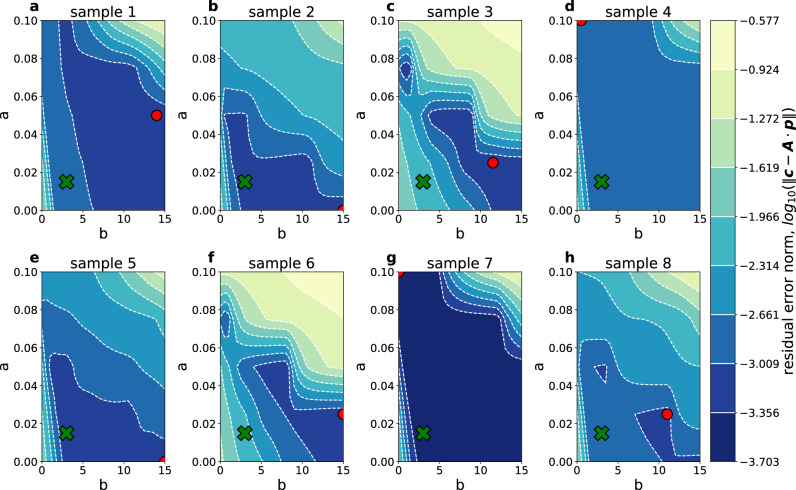
Parametrization of the frequency factor. Screening of parameters *a* (y-axis) and *b* (x-axis) for the kinetic compensation effect (Equation (3)) of the frequency factor for all samples 1 to 8 in **a**–**h**, respectively. The chosen parameter set *a* = 0.015 and *b* = 3.0 is marked by a green cross, whereas the red circle displays the parameter set obtained from an individual optimization of the frequency factor for each sample.

How the frequency factor *ω* should best be determined is an ongoing discussion, as multiple combinations of *ω* and *p*(*E*) might fit almost equally well to experimental data^13,16,17,20,21,28,29^. The typical choices for the frequency factor are either a temperature-dependent form with parameters *ω*_*o*_ and *m*,

2 $$\omega ={\omega }_{0}{T}^{m},$$

or the kinetic compensation effect

3 $$lo{g}_{10}\omega =aE+b,$$

with parameters *a* and *b* describing the activation energy dependence of *ω*^12,13,16,17,20,21^. The choice of *a* and *b* approximately affects the broadness and mean value of the *p*(*E*) distribution, respectively. This is crucial for the quantitative interpretation of the *fingerprint* distribution *p*(*E*), but does not change its qualitative distribution^16^. Here we follow the kinetic compensation effect (Numerical implementation in Supplementary Equation 1) and fix the parameters to *a* = 0.015 and *b* = 3.0 (green cross in Fig. 7a to h), resulting in a frequency factor of *ω*(*E*) = 10^0.015⋅*E*+3.0^ *s*^−1^ which yields reasonable detrapping energy values in the *p*(*E*) distribution and acceptable residual errors when scanning through a number of combinations for *a* and *b*, as suggested by Hemingway et al.^16^ (see Fig. 7).

Evidently, large regions of consistently small residual errors or the occurrence of multiple minima within the parameter search space (e.g., in Fig. 7c, f, h) make a rigorous determination of the frequency factor a challenging task. Though the analysis based on residual errors of the forward modeled retained hydrogen fraction, as done in Fig. 6 and Fig. 5, qualitatively yields similar results even if the frequency factor is separately optimized and chosen for each TDS spectrum (i.e., the red circles in Fig. 7a–h), the underlying calculated *fingerprints* are completely different and inconsistent. While this might be insignificant for the type of analysis conducted in section 2.3 (i.e., the sorting of TDS spectra according to alloy type), a consistent approach for interpreting the *fingerprint* spectra (as in Fig. 4) will be crucial for future application in TDS interpretation. Methods to rigorously determine the frequency factor are currently being tested, however, a conclusion is yet to be reached. Nonetheless, the purpose of the present investigation is to showcase the sensitivity of the proposed *fingerprint* method for TDS interpretation and its potential benefits to the field. In that, the *fingerprint* method succeeded by correctly identifying TDS spectra belonging to three model Fe-C alloys with increasing C content.

The second aforementioned problem, which is the noise-sensitivity of the model, stems from finding *p*(*E*) as the inverse solution to Equation (24) or Equation (25)^15,16^. Finding this inverse is an ill-posed problem, where even small changes in the measurement data reflected in ***c*** may drastically alter the obtained solution ***p*** (i.e. *p*(*E*))^12,15,16,30^. In order to alleviate the noise-sensitivity, Tikhonov regularization^31^ is commonly employed to seek a minimally complex, i.e. “rough”, solution *p*(*E*) while retaining solution accuracy^12,14–16,30^. Considering the solution roughness (Supplementary Equation 2) in the constrained minimization problem (Equation (25)) leads to

4 $$\mathop{\min }\limits_{{\boldsymbol{p}}}\left\{\left\Vert {\boldsymbol{c}}-{\boldsymbol{A}}\cdot {\boldsymbol{p}}\right\Vert +\lambda \left\Vert {\boldsymbol{R}}\cdot {\boldsymbol{p}}\right\Vert \right\},$$

where *λ* is a scalar regularization parameter balancing the relative weights of the solution roughness and residual error norm^14–16^.

**Fig. 8:**
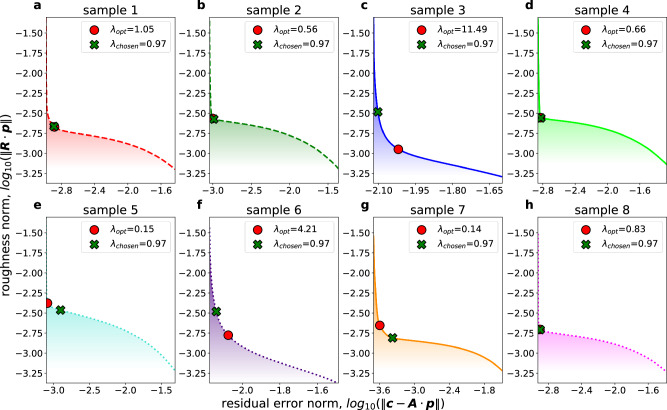
L-curves for all samples. **a**–**h** display the L-curves of individual samples 1 to 8, respectively. The point of maximum curvature *λ*_*opt*_ for each sample is marked by a red circle, whereas the chosen value for the regularization parameter *λ* = 0.97 is marked by a green cross.

The optimal choice for *λ* is not known a priori and is typically determined by the so-called “L-curve”, where the roughness norm $\left\Vert {\boldsymbol{R}}\cdot {\boldsymbol{p}}\right\Vert$ is plotted against the residual error norm $\left\Vert {\boldsymbol{c}}-{\boldsymbol{A}}\cdot {\boldsymbol{p}}\right\Vert$ on a double-logarithmic scale for *λ*-values spanning several orders of magnitude^12,14–16,30^. Such a plot typically displays an “L”-like shape and the optimal choice of *λ* is reasoned to be the corner, i.e., the point of maximum curvature, of that curve. At the upper left branch of the curve, where *λ* < *λ*_*opt*_, the solution roughness is strongly increased with little improvement to the residual error. On the contrary, the residual error increases drastically with only limited changes in solution roughness at the lower right branch of the “L” where *λ* > *λ*_*opt*_. The right choice of *λ*_*opt*_ (red circle in Fig. 8a–h) optimally provides the most simple solution that fits the data to the point where the residual error is approximately equal to the noise level^12,14,30^.

The L-curves for the investigated TDS spectra (sample 1 to sample 8) are displayed in Fig. 8a–h, respectively. The regularization parameter *λ* is chosen to be 0.97 for all spectra (green cross in Fig. 8a–h), thus offering a consistent approach that constitutes a compromise for all investigated cases.

In conclusion, by adopting the distributed activation energies model, we propose a robust way to get an insight into material microstructure features relevant for hydrogen trapping. As we have demonstrated, the proposed *fingerprint* method is not only capable of predicting the number of different trap types consistent with classical methods, but for the considered cases, it is also able to do so with only a single heating rate in theory, thus marking a potential three to four times higher experimental efficiency. Furthermore, rather than attributing a single effective hydrogen detrapping energy to each trap, the *fingerprint* calculates an energy spectrum reflective of the nuances in hydrogen trapping for each trap type. While exact quantification of the *fingerprint* is yet to be performed, the obtained energies of the individual trap peaks already closely match those from conventional analysis. However, even without a detailed interpretation, the method offers a robust quantitative metric for comparing TDS spectra, irrespective of the heating rate at which they are measured.

The *fingerprint* method continues a tradition of cross-disciplinary innovation set by Choo and Lee in 1982, who revolutionized hydrogen analysis by applying Kissinger’s theory for analysis of hydrogen TDS. This method not only updates the current state-of-the-art methodology for TDS spectra interpretation but also provides a way to enhance the efficiency and accuracy of experimental investigations. The ability to obtain material-specific energy fingerprints from a single heating rate marks a substantial improvement over the traditional Choo-Lee analysis, which requires multiple heating rates. In addition to increased efficiency, the *fingerprint* method has the potential to determine activation energies for other hydrogen-releasing processes that do not conform to classical detrapping reactions, such as austenite transformation. Furthermore, the *fingerprint* as a spectrum of trapping energies provides a new form of input for continuum simulations of hydrogen diffusion and embrittlement. The method exemplifies the ongoing evolution of scientific techniques, drawing on synergies between diverse fields to advance our knowledge in the field of hydrogen-microstructure interaction. This approach promises to bring new insights and methodologies to the hydrogen sector, continuing the legacy of innovation established by Choo and Lee.

## Methods

### *Fingerprint* model

In order to study the reaction rate of thermal dissociation of minerals during differential thermal analysis, Kissinger^3^ proposed a kinetic equation in the form of

5 $$\frac{dx}{dt}=\omega {\left(1-x\right)}^{y}\,\exp \left(-\frac{{E}_{a}}{RT}\right),$$

where *x* is the reacted fraction, *dx*/*dt* the reaction rate, *ω* is a frequency factor, *y* the reaction order, *E*_*a*_ the reaction activation energy, *R* the ideal gas constant, and *T* the absolute temperature. This generalized kinetic equation was later adapted by Choo and Lee^1^ and introduced to the field of hydrogen trapping, assuming a first order kinetic reaction (*y* = 1)^2^. Setting $x=\left({c}_{H,i}^{0}-{c}_{H,i}\right)/{c}_{H,i}^{0}$^1^ transforms Equation (5) to

6 $$\frac{d{c}_{H,i}}{dt}=-\omega \,{c}_{H,i}\,\exp \left(-\frac{{E}_{a,i}}{RT}\right),$$

where *c*_*H*,*i*_ is the amount of hydrogen trapped in a trapping site *i* at time *t*, ${c}_{H,i}^{0}$ is the initial amount of hydrogen trapped in that site (${c}_{H,i}^{0}={c}_{H,i}(t=0)$), and *E*_*a*,*i*_ is the corresponding detrapping activation energy. For traps releasing hydrogen directly via the host metal lattice, *E*_*a*,*i*_ fulfills the Kirchheim criterion *E*_*a*,*i*_ ≅ *E*_*b*,*i*_ + *E*_*m*_^2,6,32^, where *E*_*b*,*i*_ is the binding energy of hydrogen to a trap *i* and *E*_*m*_ is the migration energy barrier of the metal lattice.

This first-order reaction process can be described with the help of the distributed activation energy model (DAEM) used to describe, for example, sulfate reduction rates^19^, the serial oxidation of organic matter^13,16,18^, and other kinetic processes^11–15,17,20^. Adopting the terminology used by Hemingway et al.^16^ to hydrogen and denoting time dependencies explicitly, Equation (6) can be split into

7 $$\frac{d{c}_{H,i}}{dt}=-{k}_{i}(t)\,{c}_{H,i}(t),$$

where the rate coefficient *k*_*i*_(*t*) follows the Arrhenius equation

8 $${k}_{i}(t)=\omega \,\exp \left(-\frac{{E}_{a,i}}{RT(t)}\right)$$

and the temperature is related to time by a constant heating rate *ϕ* according to *T* = *T*_0_ + *ϕt*, with *T*_0_ as the start temperature of the measurement. Integrating Equation (6) from *t* = 0 to *t* yields the hydrogen concentration remaining in trap *i* at time *t*

9 $${c}_{H,i}(t)={c}_{H,i}(0)\,\exp \left[-{\kappa }_{i}(t)\right],$$

where the time-integrated decay coefficient *κ*_*i*_(*t*) is

10 $${\kappa }_{i}(t)=\omega \mathop{\int}\nolimits_{0}^{t}\exp \left[-\frac{{E}_{a,i}}{RT(t^{\prime} )}\right]dt^{\prime} .$$

Assuming independent detrapping kinetics for a number of *n* different traps with respective distinct activation energies *E*_*a*,*i*_, the overall detrapping kinetics behave as a superposition of the individual traps^13–17,19,20^. In other words, the total hydrogen concentration *c*_*H*,*tot*_(*t*) remaining in the TDS sample at time *t* is the sum of the hydrogen concentrations *c*_*H*,*i*_(*t*) retained in each trap *i*, respectively:

11 $${c}_{H,tot}(t)=\mathop{\sum }\nolimits_{i = 1}^{n}{c}_{H,i}(t).$$

Here, interstitial lattice positions are considered as hydrogen “traps” with an activation energy *E*_*a*,*i*_ equal to the migration energy barrier *E*_*m*_ (≈ 5 to 9 kJ/mol for bcc Fe^2,6,32,33^). Similarly, the initial hydrogen concentration ${c}_{H,tot}^{0}={c}_{H,tot}(t=0)$ in the TDS sample equals the sum of hydrogen concentrations in each trap:

12 $${c}_{H,tot}^{0}=\mathop{\sum }\nolimits_{i = 1}^{n}{c}_{H,i}(0).$$

Then, the initial fraction of hydrogen trapped in trap *i* is calculated as

13 $${p}_{i}=\frac{{c}_{H,i}(0)}{{c}_{H,tot}^{0}},$$

where the individual trap fractions *p*_*i*_ of all traps must sum up to unity:

14 $$\mathop{\sum }\nolimits_{i = 1}^{n}{p}_{i}=1.$$

Inserting Equation (9) and Equation (13) into Equation (11) results in the time-evolution of the hydrogen fraction in the whole sample, providing the link to the measured TDS spectrum:

15 $$g(t)\equiv \frac{{c}_{H,tot}(t)}{{c}_{H,tot}^{0}}=\mathop{\sum }\nolimits_{i = 1}^{n}{p}_{i}\,\exp \left[-{\kappa }_{i}(t)\right].$$

So far, this model displays the same limitation as the Gaussian deconvolution of TDS spectra or their interpretation using diffusion models for simulation, that is the presumption of the number of traps *n* and, in many cases, their respective detrapping energy *E*_*a*,*i*_^2^. However, a transformation from a finite number of traps *n* with detrapping energy *E*_*a*,*i*_ to a model where detrapping energies *E* vary over a continuous, non-negative spectrum can be straightforwardly made to alleviate this shortcoming^16^. Thereby, variables *c*_*H*,*i*_(*t*), *k*_*i*_(*t*), *κ*_*i*_(*t*), *p*_*i*_, and *E*_*a*,*i*_ in Equation (6) to Equation (15) associated with a distinct trap *i* are replaced by their respective continuous counterparts *c*_*H*_(*t*, *E*), *k*(*t*, *E*), *κ*(*t*, *E*), *p*(*E*), and *E*^16^. Also, all sums over the number of traps are replaced by integrals over the continuous, non-negative energy spectrum:

$$\mathop{\sum }\nolimits_{i = 1}^{n}\to \mathop{\int}\nolimits_{0}^{\infty }dE.$$

This transformation yields for Equation (15)

16 $$g(t)=\mathop{\int}\nolimits_{0}^{\infty }p(E)\,\exp \left[-\kappa (t,E)\right]\,dE,$$

with the decay coefficient *κ*(*t*, *E*) from Equation (10)

17 $$\kappa (t,E)=\omega \mathop{\int}\nolimits_{0}^{t}\exp \left[-\frac{E}{RT(t^{\prime} )}\right]dt^{\prime} ,$$

and the initial hydrogen fraction *p*(*E*) and hydrogen concentration *c*_*H*_(*E*) in a trap of energy *E* from Equation (13),

18 $$p(E)=\frac{{c}_{H}(E)}{{c}_{H,tot}^{0}}.$$

For the numerical implementation, the right-hand side of the governing Equation (16) for the time-evolution of the hydrogen fraction within the entire TDS sample can be split into the initial fraction *p*(*E*) of hydrogen residing in a trap of detrapping energy *E* and the corresponding exponent of the time-integrated decay coefficient *κ*(*t*, *E*) (see Equation (17))^16^. The goal is to find the unknown initial fractional distribution of hydrogen *p*(*E*) over the continuous energy range *E*, which we call the trapping energy *fingerprint*, as the inverse numerical solution to Equation (16), for which we follow the implementation of Hemingway et al.^16^.

As the term associated with the decay coefficient *κ*(*t*, *E*) is only model-dependent, we can discretize this part of Equation (16) as a matrix ***A*** with entries

19 $$\begin{array}{rcl}{A}_{jl}&=&\exp \left\{-\mathop{\sum }\limits_{u = 1}^{j}\omega \exp \left[-\frac{{E}_{l}}{RT({t}_{u})}\right]\Delta {t}_{u}\right\}\Delta E,\\ j&=&1,\ldots ,{n}_{t},\\ l&=&1,\ldots ,{n}_{E}.\end{array}$$

This matrix ***A***, also referred to as the “design matrix”, represents the time and energy discretization of the decay coefficient *κ*(*t*, *E*). Thereby, *t*_*j*_ and *E*_*l*_ loop through the time and energy vectors ***t*** and ***E***, respectively. The measurement time is discretized into the vector ***t*** with *n*_*t*_ entries according to:

20 $$\begin{array}{rcl}\Delta {t}_{j}&=&\frac{1}{2}\left({t}_{j+1}-{t}_{j-1}\right),\\ j&=&2,\ldots ,{n}_{t}-1.\end{array}$$

In order to account for the validity of the used times, the *t*_*j*−1_ and *t*_*j*+1_ is replaced by *t*_*j*_ at the start (*j* = 1) and the end (*j* = *n*_*t*_), respectively.

Since it is not feasible to integrate over an infinite energy space, the energy vector ***E*** is constrained within the bounds of *E*_*min*_ and *E*_*max*_ with a number of *n*_*E*_ nodes used for discretization:

21 $$\Delta E=\frac{{E}_{max}-{E}_{min}}{{n}_{E}}.$$

Next, the left-hand side of Equation (16), representing the fraction of hydrogen retained in the whole TDS sample at time *t*, can be inferred from the measured TDS spectra as:

22 $$\frac{{c}_{H,tot}(t)}{{c}_{H,tot}^{0}}=1-\frac{1}{{c}_{H,tot}^{0}}\mathop{\int}\nolimits_{0}^{t}{\Phi }_{H}(t^{\prime} )\,dt^{\prime} ,$$

where *Φ*_*H*_(*t*) is the measured hydrogen outflux at time *t* in the TDS spectrum. The function obtained in Equation (22) is interpolated to each *t*_*j*_ within the time vector ***t*** to obtain the discretized vector ***c***. Note that vectors ***t*** and ***c*** share the same number of nodes *n*_*t*_.

Lastly, the unknown initial hydrogen distribution *p*(*E*) is discretized into the vector ***p*** with *n*_*E*_ nodes and entries:

23 $$\begin{array}{rcl}{p}_{l}&=&\frac{1}{\Delta E}\mathop{\int}\nolimits_{{E}_{l}-\frac{1}{2}\Delta E}^{{E}_{l}+\frac{1}{2}\Delta E}p(E)dE,\\ l&=&1,\ldots ,{n}_{E}.\end{array}$$

Thus, Equation (16) can be reformulated as a matrix equation reading as

24 $${\boldsymbol{c}}={\boldsymbol{A}}\cdot {\boldsymbol{p}}.$$

As any fractional hydrogen occupation must naturally satisfy 0≤*p*_*l*_≤1, simply finding ***p*** by multiplying ***c*** with ***A***^−1^ may lead to nonphysical values of *p*_*l*_ < 0. Therefore, the calculation of ***p*** is based on the following minimization

25 $$\mathop{\min }\limits_{{\boldsymbol{p}}}\left\Vert {\boldsymbol{c}}-{\boldsymbol{A}}\cdot {\boldsymbol{p}}\right\Vert \equiv {\left[\mathop{\sum }\limits_{j = 1}^{{n}_{t}}{\left({c}_{j}-\mathop{\sum }\nolimits_{l = 1}^{{n}_{E}}{A}_{jl}{p}_{l}\right)}^{2}\right]}^{\frac{1}{2}},$$

constrained by the fact that the fraction of hydrogen in a trap cannot be negative

26 $${p}_{l}\ge 0,\,l=1,\ldots ,{n}_{E}$$

and that all individual fractions must sum to unity (see also Equation (14))

27 $$\mathop{\sum }\nolimits_{l = 1}^{{n}_{E}}{p}_{l}=1,\,l=1,\ldots ,{n}_{E}.$$

The constrained minimization described by Equation (25) to Equation (27) is implemented in the code of Hemingway et al.^16^, making use of the nonnegative least squares algorithm developed by Lawson and Hanson^34^ contained within the Python (3.9.18) package SciPy (1.11.4)^27^.

In order to verify solution accuracy and apply *p*(*E*) to predict TDS spectra for other combinations of total hydrogen concentration ${c}_{H,tot}^{0}$ and heating rate *ϕ*, the time evolution of either the retained hydrogen fraction ***c*** as the discretized form of ${c}_{H,tot}(t)/{c}_{H,tot}^{0}$ (see Equation (22)) or the hydrogen flux directly as *Φ*_*H*_ = − *dc*_*H*,*tot*_(*t*)/*dt* need to be calculated. The former method is the simplest, as the estimated time evolution of retained hydrogen fraction ***c***_*est*._ can be straightforwardly calculated from the found regularized solution ***p*** ≡ *p*(*E*) and the design matrix for the new heating rate ***A***_*ϕ*_ with otherwise identical parameters according to Equation (24) as^16^:

28 $${{\boldsymbol{c}}}_{est.}={{\boldsymbol{A}}}_{\phi }\cdot {\boldsymbol{p}}.$$

The estimated hydrogen flux *Φ*_*H*,*est*._(*t*) over time (or temperature *Φ*_*H*,*est*._(*T*)), i.e. the TDS spectrum, is then given by:

29 $${\Phi }_{H,est.}(t)=-\frac{d{{\boldsymbol{c}}}_{est.}}{dt}\cdot {c}_{H,tot}^{0},$$

which requires differentiation of the estimated retained fraction ***c***_*est*._ with respect to time (as discretized time vector ***t***), resulting in a noisy flux.

Here, we slightly deviate from the code as implemented by Hemingway et al.^16^ in order to alleviate this problem. By inserting Equation (28) into Equation (29), it is immediately visible that

30 $${\Phi }_{H,est.}(t)=-\frac{d\left({{\boldsymbol{A}}}_{\phi }\cdot {\boldsymbol{p}}\right)}{dt}\cdot {c}_{H,tot}^{0}=-\left(\frac{d{{\boldsymbol{A}}}_{\phi }}{dt}\cdot {\boldsymbol{p}}\right)\cdot {c}_{H,tot}^{0},$$

where the time-derivative of the design matrix can be written as

31 $$\frac{d{{\boldsymbol{A}}}_{\phi }}{dt}=-\omega \exp \left(-\frac{E}{RT(t)}\right)\exp \left[-\omega \mathop{\int}\nolimits_{0}^{t}\exp \left(-\frac{E}{RT(t^{\prime} )}\right)dt^{\prime} \right],$$

or

32 $$\begin{array}{lll}{\left(\frac{dA}{dt}\right)}_{\phi ,jl}\,\,=\,-{\omega }_{l}\exp \left(-\frac{{E}_{l}}{RT({t}_{j})}\right)\exp \left[-{\omega }_{l}\mathop{\sum }\limits_{u = 1}^{j}\exp \left(-\frac{E}{RT({t}_{u})}\right)\Delta {t}_{u}\right]\Delta E,\\\,\,j\qquad\quad\;=\,1,\ldots ,{n}_{t},\\\,\,l\qquad\quad\;\,=\,1,\ldots ,{n}_{E},\end{array}$$

with *T*(*t*) = *T*_0_ + *ϕt* as a function of the heating rate *ϕ*. Although Equation (29) and Equation (30) are analytically equivalent, the flux calculated by inserting the derivative of the design matrix from Equation (32) into Equation (30) displays considerable lower noise compared to Equation (29) due to the numerical procedure.

### Materials and experimental methods

Three model Fe-C alloys with varying C content characterized in Refs. ^22,23^ were used to investigate the robustness of the *fingerprint* method. The materials were austenitized for 20 minutes, followed by a brine quench to room temperature, leading to a fully martensitic matrix. An overview of the C content, heat treatment parameters and microstructural features are provided in Table 1. Notably, the C content in alloy 3 was high enough to induce 12.7 vol.-% retained austenite as well as the presence of quench cracks^22,23^.

**Table 1:** Carbon content, austenitization temperature, prior austenite grain size, and hardness of the model Fe-C alloys^22^

Alloy	C content (wt.-%)	Austenitizing temperature (^∘^C)	PAG size (*μm*)	Hardness (HV3)
Fe-0.2C (alloy 2)	0.2	900	31.4 ± 2.8	421 ± 29
Fe-0.4C (alloy 1)	0.4	880	32.2 ± 7.8	672 ± 10
Fe-1.1C (alloy 3)	1.1	1000	71.0 ± 8	871 ± 17

The corresponding alloy numbers as assigned in Fig. 4 and Fig. 6 are given in brackets.

TDS measurements were conducted using a Galileo G8 setup. Samples, each with a diameter of 20 mm and a thickness of 1 mm, were charged in a 0.5 M H_2_SO_4_ solution containing 1 g/L thiourea at a current density of 0.8 mA/m^2^ for 2 h. This charging duration was determined to be sufficient for achieving hydrogen saturation in the samples. Post charging, the specimens were meticulously cleaned and dried before being placed in the TDS setup. The interval between the end of charging and the initiation of the TDS signal was approximately 1 minute. For alloys 1 and 2, three different heating rates were employed: 0.17 K/s, 0.25 K/s, and 0.33 K/s. Alloy 3 was subjected to two heating rates: 0.17 K/s and 0.33 K/s.

Importantly, experiments were carried out by the research group of Ghent University and experimental details were a priori unknown to the group employing the *fingerprint* method to analyze the TDS spectra.

## Supplementary information

Supplementary information

## Data Availability

The raw data related to this manuscript is available upon request to P.H. and T.D.
