# Supplementary material for: The fingerprint method for analysis of thermal desorption spectra
Source: Npj Mater Degrad. 2025 Dec 10;10(1):7. doi: 10.1038/s41529-025-00718-z (PMC12799485; doi:10.1038/s41529-025-00718-z)
Supplement: Supplementary file 1 — Supplementary information [file 41529_2025_718_MOESM1_ESM.pdf]

The Fingerprint method for analysis of thermal desorption spectra

Philipp Hammer[1*†], Oleg E. Peil[2†], Ahmad Azizpour[2], Liese Vandewalle[3], Kim Verbeken[3], Tom Depover[3], Vsevolod I. Razumovskiy[1]

[1]Christian Doppler Laboratory for digital material design guidelines for mitigation of alloy embrittlement, Materials Center Leoben Forschung GmbH, Vordernberger Straße 12, Leoben, 8700, Austria.
[2]Materials Center Leoben Forschung GmbH, Vordernberger Straße 12, Leoben, 8700, Austria.
[3]Department of Materials, Textiles and Chemical Engineering, Ghent University, Technologiepark 46, Zwijnaarde, 9052, Belgium.

*Corresponding author(s). E-mail(s): philipp.hammer@mcl.at;
Contributing authors: oleg.peil@mcl.at; ahmad.azizpour@jku.at; Liese.Vandewalle@UGent.be; kim.verbeken@ugent.be; tom.depover@ugent.be; vsevolod.razumovskiy@mcl.at;
[†]These authors contributed equally to this work.

Supplementary information

1 Kinetic compensation effect

In this work, we employ an energy-dependent frequency factor according to Equation 3, where $\boldsymbol{\omega}$ is a vector of length n_E interpolated onto the nodes of the energy vector $\boldsymbol{E}$,

which transforms Equation 19 to:

$$A_{jl} = \exp\left\{-\sum_{u=1}^{j}\omega_l \exp\left[-\frac{E_l}{RT(t_u)}\ \Delta t_u\right]\right\}\Delta E\ , \qquad (1)$$
$$j = 1, ..., n_t\ ,$$
$$l = 1, ..., n_E\ .$$

2 Tikhonov regularization

For the Tikhonov regularization, the solution roughness is represented by the norm of the first derivative of the solution vector $\boldsymbol{p}$ [3, 4]

$$\left\|\frac{dp(E)}{dE}\right\| = \left[\sum_{l=2}^{n_e-1}\left(\frac{p_{l+1}-p_l}{\Delta E}\right)^2\right]^{\frac{1}{2}} \equiv \|\boldsymbol{R}\cdot\boldsymbol{p}\|\ , \qquad (2)$$

where the derivative is discretized by the bi-diagonal first derivative operator matrix $\boldsymbol{R}$ [1–3]. As $\boldsymbol{p}$ is zero outside the valid range $E_{min} < E < E_{max}$ of the energy vector, the first row of $\boldsymbol{R}$ is set to $[1\ \mathbf{0}]$ and an additional last row of $[\mathbf{0}\ \ -1]$ is added, where $\mathbf{0}$ is a zero vector with length $n_E - 1$ and $\boldsymbol{R}$ displays final dimensions of $[n_E + 1 \times n_E]$ [1, 3].

References

[1] Forney, D. C. & Rothman, D. H. Inverse method for estimating respiration rates from decay time series. *Biogeosciences* **9**, 3601–3612 (2012). URL http://dx.doi.org/10.5194/bg-9-3601-2012.

[2] Forney, D. C. & Rothman, D. H. Common structure in the heterogeneity of plant-matter decay. *Journal of the Royal Society Interface* **9**, 2255–2267 (2012). URL http://dx.doi.org/10.1098/rsif.2012.0122.

[3] Hemingway, J. D., Rothman, D. H., Rosengard, S. Z. & Galy, V. V. Technical note: An inverse method to relate organic carbon reactivity to isotope composition from serial oxidation. *Biogeosciences* **14**, 5099–5114 (2017). URL http://dx.doi.org/10.5194/bg-14-5099-2017.

[4] Tikhonov, A. N. & Arsenin, V. Y. *Solutions of ill-posed problems* (V. H. Winston & Sons, Washington, D.C.: John Wiley & Sons, New York, 1977).
